# Autism: Comorbidities and Treatment Patterns in the Real World, a Retrospective Cohort Study Among Children, Adolescents and Adults Newly Diagnosed with Autism

**DOI:** 10.1007/s10803-021-05289-x

**Published:** 2021-10-08

**Authors:** Azza Shoaib, M. Soledad Cepeda, Gayle Murray, Rachel Ochs-Ross

**Affiliations:** Janssen Research & Development, LLC, 1125 Trenton-Harbourton Road, Titusville, NJ 08560 USA

**Keywords:** Comorbid neurological conditions, Real world data, Intervention patterns, Treatment intervention

## Abstract

We used real world data to summarize comorbidities and interventions among patients newly diagnosed with autism spectrum disorder (ASD). Data were derived from two claims-based US healthcare databases; Medicaid and Optum to construct a retrospective cohort of 36,000 patients. Attention-Deficit-Hyperactivity-Disorder (ADHD) was the most common co-morbidity (Medicaid: 50.09%; Optum: 44.16%), followed by mood disorder (Medicaid: 16.56% and Optum: 17.47%). Most patients received at least one type of treatment. Behavioral therapy was common (74.64% in Medicaid and 71.97% in Optum). More than half the cohorts received at least 1 pharmacotherapy. However, pharmacotherapies were diverse. Combination therapy and therapy switching was common. Understanding the clinical diversity and complexity of patients with ASD is an important first step in understanding unmet therapeutic needs.

**Supplementary Information:**

The online version contains supplementary material available at 10.1007/s10803-021-05289-x.

## Introduction

In the US, about 1 in 59 children has been identified with autism spectrum disorder (ASD) (Xu et al., 2018). Studies in Asia, Europe, and North America have identified individuals with ASD with an average prevalence between 1% and 2% (Onaolapo & Onaolapo, 2017; Qiu et al., 2020). Moreover, studies have demonstrated that the incidence of autism symptoms and clinical diagnosis have increased over the past decades in the United States and elsewhere (Myers et al., 2019). A great variety of comorbidities and prognoses are hallmarks of ASD (Masi et al., 2017).

The key features of ASD include (1) atypical social communication and (2) Repetitive patterns or behavior, interests, or activities. In addition, patients frequently have an array of comorbidities and are often exposed to a wide range of interventions (Masi et al., 2017). However, the full picture of comorbidity burden and intervention utilization, including what treatments are first used, in what combination, and changes in the therapy have not been well characterized in the literature. Observational data provide a unique opportunity to characterize patients with ASD, their comorbidities and the treatments and treatment patterns they receive in the real world.

In this study we use data from two large national claims systems to examine the occurrence of comorbid neurological and psychological conditions among patients newly diagnosed with ASD in the United States. We also explore and summarize temporal patterns of behavioral and pharmacological intervention through the first 4 lines of therapy received post diagnosis.

Understanding the current landscape of both treatment and comorbidities provides a meaningful reference to help understand this vulnerable population, the limitations of current therapies and provide information to help guide development of new therapies. While this analysis is purely descriptive, a priori we hypothesized that patients with ASD will have a high proportion of coexisting comorbidities and that a high variation in treatment pattern will be observed.

## Methods

We used data from two large US healthcare databases. IBM MarketScan® Multi-State Medicaid Database (MDCD) and Optum® De-Identified Clinformatics® Data Mart Database—Date of Death (Optum)) to construct a retrospective cohort study.

### Identification of ASD Cohort

We identified patients newly diagnosed with ASD in the two large national insurance claims data bases during January of 2015 through December of 2016. To capture current treatment utilization and practices, we focused on the most recent 5 years of data. We adopted Burke et al., (2014) algorithm to define patients with ASD. This algorithm has a reported positive predictive value of 87.4%. Patients were included if they had two diagnoses of ASD made on or after 1 January 2015, where the two diagnosis were within 1 year of each other, had continuous observation of at least 180 days prior the first ASD diagnosis and have 3 years of observation after the first ASD diagnosis. ASD was defined using the following ICD10CM codes: F84.9, F84.5, F84.0, F84.8. Requiring two outpatient diagnoses within a year would likely lead to improvements in specificity but a reduction in sensitivity of the deffinition. This is a trade-off we deliberately made to improve certainty that we only included true ASD patients. Only patients with an incident diagnosis of ASD were included. Patients were followed for 3 years after they enter the cohort.

### Data Sources

Two sources of claims based observational data were used for this analysis, IBM MarketScan® Multi-State Medicaid Database (MDCD) and Optum® De-Identified Clinformatics® Data Mart Database—Date of Death (Optum). MDCD is a curation of adjudicated US health insurance claims for Medicaid enrollees from 10 to 12 states and includes hospital discharge diagnoses, outpatient diagnoses and procedures, and outpatient pharmacy claims. This licensed database has no information on which states are included. Optum is an adjudicated US administrative health claims database for members of private health insurance across the country, who are fully insured in commercial plans or in administrative services only (ASOs), Legacy Medicare Choice Lives (prior to January 2006), and Medicare Advantage (Medicare Advantage Prescription Drug coverage starting January 2006). The Optum population is primarily representative of commercial claims patients (0–65 years old) with some patients covered by Medicare (65 + years old). The major data elements contained within this database are outpatient pharmacy dispensing claims (coded with National Drug Codes (NDC), inpatient and outpatient medical claims which provide procedure codes (coded in CPT-4, HCPCs, ICD-9-CM or ICD-10-PCS) and diagnosis codes (coded in ICD-9-CM or ICD-10-CM).

The two databases have been harmonized into the Observational Medical Outcomes Partnership (OMOP) Common Data Model (CDM) version 5.3 to facilitate analyses. The treatment sequence analysis was performed within the Observational Health Data Sciences and Informatics (or OHDSI, pronounced “Odyssey”) framework. Use of the common data model allowed for consistent and precise replication across each of the two databases (Hripcsak et al., 2015). The use of the IBM MarketScan and Optum claims databases was reviewed by the New England Institutional Review Board (IRB) and was determined to be exempt from broad IRB approval, as this research project did not involve human subjects’ research.

### Baseline Characteristics

To describe the baseline characteristics of patients with ASD included in the study, we identified medical conditions in the databases present six months before or at the index date, which was the date of the first ASD diagnosis, using the systematized Nomenclature of Medicine‐Clinical Terms (SNOMED CT). SNOMED is a standardized, multilingual vocabulary of clinical terminology that is used by physicians and other healthcare providers for the electronic exchange of clinical health information (Reich et al., 2012). The SNOMED classification allows mapping of various diagnostic languages, including ICD-10-CM, to a single standardized set of concepts, and is used by the CDM leveraged for the present study. We report the prevalence proportion of most common medical conditions stratified by sex and 3 age groups (0–9 years, 10–17 years, 18 and above).

### Treatments and Treatment Sequencing

We considered 3 main categories of treatments: (a) behavioral therapy; which included psychosocial therapy, family therapy, applied behavior therapy, specialized therapy such as speech, occupational and others- the full list of procedures with the corresponding SNOMED and CPT4 is provided in supplementary table 2.; (b) therapeutic brain stimulation approaches, which included transcranial magnetic stimulation (TMS) and transcranial direct current stimulation, and (c) pharmacotherapy. For pharmacotherapy, we included psychotropic and other medications used to treat targeted symptoms associated with ASD based on prior literature. Medication patterns were captured at the class level and included selective serotonin reuptake inhibitors (SSRIs), serotonin and norepinephrine reuptake inhibitors (SNRIs), tricyclic antidepressants (TCAs), monoamine oxidase inhibitors (MAOIs), other antidepressants (including bupropion and trazodone, among others), anxiolytics, antipsychotics, atypical antipsychotics (including risperidone and aripiprazole), anticonvulsants, centrally acting sympathomimetics (including methylphenidate and amphetamine), hypnotics and sedatives, NMDA receptor antagonists (including memantine and amantadine), imidazoline receptor agonists (including guanfacine and clonidineand). In addition, single drugs that were reported to be individually used by ASD patients, such as acetylcysteine, pentoxifylline, riluzole, celecoxib, simvastatin and baclofen were considered. The full list of medications and their corresponding RxNorm ingredient codes (which is a standardized drug nomenclature linking system that use different vocabularies) can be found in the supplementary table 3*.*

Treatment sequences were captured during 30 days prior to ASD diagnosis and through 3 years of follow up. The term “treatment line” is used to describe the sequence of therapies and combinations of therapies during this time. Use of a specific therapy was captured at the first instance and not counted again in later lines of therapy—for example an individual filling a SNRI switching to a SSRI, and then moving back to a SNRI would only be captured as switching from a SNRI to SSRI. For medications captured at the class level, in-class switching, and in-class combination therapy was not captured. For pharmacotherapy, drug exposure duration was calculated as the time from the first fill for the drug (or a drug in a medication class for drugs captured on the class level) until discontinuation of that medication, allowing for gaps of up to 30 days beyond the days’ supply of a prescription. Combination therapy with multiple treatments was defined as having at least 30 days of overlap of more than one treatment. A fill for a medication or a start of a therapeutic procedure following discontinuation of a previous treatment or with fewer than 30 days of overlap was considered a switch. All analyses were done using ATLAS, an open-source application developed as a part of the Observational Health Data Sciences and Informatics (OHDSI), intended to provide a unified interface to patient level data and analytics (https://github.com/OHDSI/Atlas/wiki).

## Results

We identified a total of 36,000 ASD patients in the two data bases (Table 1). In MDCD data, we identified 29,694 patients with mean age of 14.09 years at index date; 76.27% were males and 23.06% were black. In Optum data, 6719 patients were identified with a mean age of 15.04 years old and 76.28% were males. Information on race is not available in Optum.

**Table 1:** ASD Cohort characteristics by data source

Characteristics	IBM marketScan® multi-state medicaid database (MDCD)		Optum® de-identified clinformatics®	
	N	%	N	%
Total	29,694	100	6719	100
Age (years) mean. Std	14.09 (12.17)		15.04 (14.90)	
0–5	5806	19.55	1388	20.66
5–9	7706	25.95	1552	23.10
10–14	5763	19.41	1414	21.09
15–17	2411	8.12	752	11.19
18 and above	8008	26.97	1610	23.96
Race				
White	17,169	57.82		
Black	6847	23.06		
Unknown race	5678	19.12%		
Gender				
Male	22,648	76.27	5125	76.28
Female	7046	23.73	1594	23.72
Index year				
2015	12,561	42.30	2,246	33.43
2016	17,133	57.70	2,469	36.75
2017	0	–	2,004	29.83
Charlson index (mean, STD)	0.36 (1.23)		0.46 (1.38)	
Treatments				
Any treatment	25,978	87.5%	5837	86.9%
Behavioral therapy	22,165	74.64%	4836	71.97%
Any Pharmacotherapy	18,430	62.1%	3951	58.8%
Centrally acting sympathomimetics	7494	25.24%	1746	25.99%
Imidazoline receptor agonists	7016	23.63%	1008	15.00%
Antipsychotics	6134	20.66%	1116	16.61%
Atypical antipsychotics	5011	16.88%	811	12.07%
Selective serotonin reuptake inhibitors	4929	16.60%	1698	25.27%
Anticonvulsant	4285	14.43%	1008	15.00%
Anxiolytics (excluding BZO)	3227	10.87%	9591	14.27%
Hypnotics and sedatives	3073	10.35%	498	7.41%
Other antidepressants	2621	8.08%	553	8.23%

We report the proportion of base-line demographics and index year and treatment utilization in each data source

### ASD Comorbidities

The most common comorbidities among patients with ASD were neurological and psychological conditions (Table 2). Attention-deficit/hyperactivity disorder (ADHD) was the most common comorbidity in both data bases (50.09% in MDCD and 44.16% in Optum), followed by mood disorder (16.56% and 17.47% in MDCD and Optum respectively) and anxiety (16.56% and 24.84% in MDCD and Optum respectively). Developmental delay was observed in 14.91% of the patients in MDCD and 13.60% in Optum. Table 2 demonstrates the proportions of these selected conditions by age group. In both data bases, ADHD was observed more in adolescents (10–17 years old) than other age groups. Anxiety was frequently observed in both adolescents and adults (18 years and older). Mood disorders were mostly prevalent in adults, while developmental delays were mostly observed in young children (0–9 years old).

**Table 2:** Selected common comorbid neurological and psychological conditions diagnosed during the 6 months prior-index period, which included the index date

Conditions	Overall		0–9 years old		10–17 years old		18 and above years old	
	N	%	N	%	N	%	N	%
Optum	6719	100	2940	100	2169	100	1610	100
ADHD	2967	44.16	1219	41.46	1300	59.94	448	27.83
Anxiety	1669	24.84	317	10.78	784	36.15	568	35.28
Developmental delay	914	13.60	768	26.12	110	5.07	36	2.24
Malaise and fatigue	107	1.59	20	0.68	27	1.24	60	3.73
Mood disorder	1174	17.47	88	2.99	526	24.25	560	34.78
Seizure and Seizure disorder	431	6.41	139	4.73	129	5.95	163	10.12
MDCD	29694	100%	13,512	100	8174	100	8008	100
ADHD	14873	50.09	7342	54.34	5403	66.10	8008	26.57
Anxiety	4788	16.12	1233	9.13	1817	22.23	8008	21.70
Developmental delay	4426	14.91	3575	26.46	649	7.94	8008	2.52
Malaise and fatigue	349	1.18	90	0.67	81	0.99	8008	2.22
Mood disorder	4917	16.56	831	6.15	1852	22.66	8008	27.9
Seizure and Seizure disorder	2507	8.44	796	5.89	579	7.08	8008	14.14

We report the proportion (prevalence) of each condition stratified by age group and data source

### ASD Treatments and Sequencing

The large majority of patients in both databases (87.5% in MDCD and 86.9% in Optum) received at least one treatment during follow-up. Behavioral therapy was by far the most common treatment received, accounting for 74.64% in MDCD and 71.97% in Optum of all patients with ASD. Therapeutic brain stimulation procedures were extremely rare and observed in only 0.01% (n = 4) of the cohort in MDCD and 0.05% (n = 3) in Optum. There were 62.1% and 58.8% of patients in MDCD and Optum respectively who received at least 1 pharmacotherapy during follow up (Table 1).

In terms of pharmacotherapy treatments, there was no one class that was predominantly used as first line or in subsequent lines of treatment (Tables 3 and 4). Regardless of the line of treatment, centrally acting sympathomimetics, were the most commonly used in both data bases (16.64% on MDCD and 10.95% in Optum) followed by imidazoline receptor agonists in MDCD (9.98%) and SSRIs in Optum (15.81%).

**Table 3:** Top 10 most common treatments during each of the first four lines of therapy in IBM MarketScan® Multi-State Medicaid Database

Treatment line	Therapy/treatment	Patient count	Rank in treatment line	% of patients in treatment line
1 (n = 25,978)	Behavioral and special therapy	17,003	1	65.45
	Centrally acting sympathomimetics	2845	2	10.95
	Imidazoline receptor agonists	2592	3	9.98
	Antipsychotics	2565	4	9.87
	Other (atypical) Antipsychotics	2042	5	7.86
	Selective serotonin reuptake inhibitors	1864	6	7.18
	Anticonvulsant	1711	7	6.59
	Hypnotics and sedatives	1242	8	4.78
	Anxiolytics (excluding BZO)	1043	9	4.01
	Other antidepressants	858	10	3.30
2 (n = 16,853)	Behavioral and special therapy	6935	1	26.70
	Centrally acting sympathomimetics	4380	2	16.86
	Imidazoline receptor agonists	4129	3	15.89
	Antipsychotics	3468	4	13.35
	Other (atypical) Antipsychotics	2725	5	10.49
	Selective serotonin reuptake inhibitors	2706	6	10.42
	Anticonvulsant	2265	7	8.72
	Anxiolytics (excluding BZO)	1436	8	5.53
	Other antidepressants	1261	9	4.85
	Hypnotics and sedatives	1228	10	4.73
3 (n = 13,116)	Behavioral and special therapy	5226	1	20.12
	Centrally acting sympathomimetics	4087	2	15.73
	Imidazoline receptor agonists	3902	3	15.02
	Antipsychotics	3461	4	13.32
	Other (atypical) Antipsychotics	2687	5	10.34
	Selective serotonin reuptake inhibitors	2600	6	10.01
	Anticonvulsant	2258	7	8.69
	Other antidepressants	1360	8	5.24
	Anxiolytics (excluding BZO)	1255	9	4.83
	Hypnotics and sedatives	740	10	2.85
4 (n = 9730)	Behavioral and special therapy	3720	1	14.32
	Imidazoline receptor agonists	3325	2	12.80
	Centrally acting sympathomimetics	3297	3	12.69
	Antipsychotics	3163	4	12.18
	Other (atypical) Antipsychotics	2429	5	9.35
	Selective serotonin reuptake inhibitors	2330	6	8.97
	Anticonvulsant	2037	7	7.84
	Other antidepressants	1350	8	5.20
	Anxiolytics (excluding BZO)	1110	9	4.27
	Hypnotics and sedatives	507	10	1.95

**Table 4:** Top 10 most common treatments during each of the first four lines of therapy in Optum® De-Identified Clinformatics

Treatment line	Therapy/treatment	Patient count	Rank in treatment line	% of patients in treatment line
1 (n = 5837)	Behavioral and special therapy	3852	65.99%	1
	Centrally acting sympathomimetics	971	16.64%	2
	Selective serotonin reuptake inhibitors	923	15.81%	3
	Antipsychotics	591	10.13%	4
	Anticonvulsant	518	8.87%	5
	Imidazoline receptor agonists	447	7.66%	6
	Other (atypical) Antipsychotics	416	7.13%	7
	Other antidepressants	254	4.35%	8
	Hypnotics and sedatives	203	3.48%	9
	Anxiolytics (Excluding BZO)	193	3.31%	10
2 (n = 3527)	Behavioral and special therapy	1556	26.66%	1
	Centrally acting sympathomimetics	958	16.41%	2
	Selective serotonin reuptake inhibitors	881	15.09%	3
	Antipsychotics	589	10.09%	4
	Imidazoline receptor agonists	506	8.67%	5
	Anticonvulsant	466	7.98%	6
	Other (atypical) Antipsychotics	404	6.92%	7
	Other antidepressants	218	3.73%	8
	Hypnotics and sedatives	192	3.29%	9
	Anxiolytics (excluding BZO)	185	3.17%	10
3 (n = 2757)	Behavioral and special therapy	1088	18.64%	1
	Selective serotonin reuptake inhibitors	851	14.58%	2
	Centrally acting sympathomimetics	774	13.26%	3
	Antipsychotics	594	10.18%	4
	Imidazoline receptor agonists	491	8.41%	5
	Anticonvulsant	406	6.96%	6
	Other (atypical) Antipsychotics	396	6.78%	7
	Other antidepressants	241	4.13%	8
	Anxiolytics (excluding BZO)	178	3.05%	9
	SNRI	96	1.64%	10
4 (n = 2066)	Behavioral and special therapy	738	12.64%	1
	Selective serotonin reuptake inhibitors	694	11.89%	2
	Centrally acting sympathomimetics	581	9.95%	3
	Antipsychotics	515	8.82%	4
	Imidazoline receptor agonists	405	6.94%	5
	Anticonvulsant	386	6.61%	6
	Other (atypical) Antipsychotics	350	6.00%	7
	Other antidepressants	238	4.08%	8
	Anxiolytics (excluding BZO)	170	2.91%	9
	SNRI	91	1.56%	10

Use of more than one treatment simultaneously (combination therapy) occurred in 20% and 25.75% of all treated patients in the 1st line of therapy in MDCD and Optum respectively. Combination therapy increased as line of therapy advanced, occurring in 75% and 64.38% among treated patients in the fourth line of therapy in MDCD and Optum respectively. Table 5 illustrates the most common first line treatment regimens (single and combination) of ASD treated patients in MDCD data. The treatment mix in combination therapies was very diverse (Table 5); for example, the combination of antipsychotics and atypical antipsychotics was the most prevalent among first line treatments but counted for only 2.88% of the treated patients.

**Table 5:** First line treatments (single and combination) among ASD cohort in in IBM MarketScan® Multi-State Medicaid Database

Treatment	Patient count	% of patients in 1st treatment line
Anticonvulsant	766	2.95
Anticonvulsant + Behavioral and special therapy	146	0.56
Antipsychotics	259	1.00
Antipsychotics + other (atypical) Antipsychotics	748	2.88
Antipsychotics + Behavioral and special therapy + other (atypical) Antipsychotics	253	0.97
Centrally acting sympathomimetics	1409	5.42
Imidazoline receptor agonists	1194	4.60
Imidazoline receptor agonists + Centrally acting sympathomimetics	169	0.65
Imidazoline receptor agonists + Behavioral and special therapy	321	1.24
Selective serotonin reuptake inhibitors (SSRI)	776	2.99
Selective serotonin reuptake inhibitors (SSRI) + Behavioral and special therapy	219	0.84
Anxiolytics (excluding BZO)	513	1.97
Behavioral and special therapy	14,359	55.27
Behavioral and special therapy + Centrally acting sympathomimetics	425	1.64
hypnotics and sedatives	910	3.50
Other antidepressants	295	1.14

**Fig. 1:**
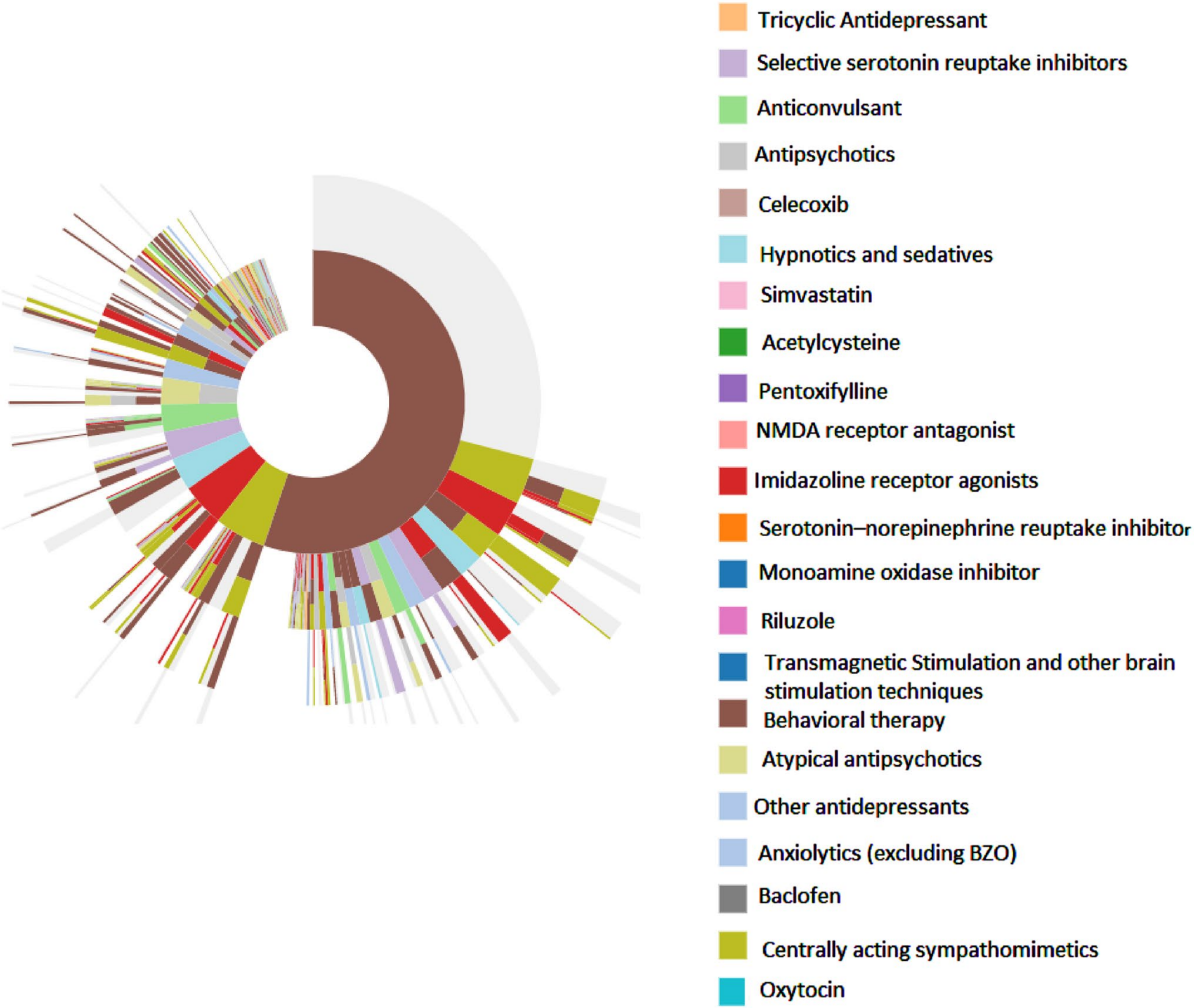
Sunburst of treatment patterns starting with first line (inner-most donut) to fourth line (outer slices) with autism spectrum disorder in MDCD. Each color represents a distinct therapy, and each layer represents a new therapy line and illustrates the sequence in which patients received different therapies; for example the large brown piece in the middle indicates first-line behavior therapy use, and the dirty dark green slice on the next outer ring adjacent to the brown indicates a switch from behavior therapy to Centrally acting sympathomimetics. Slices that have multiple colors indicate combination therapy with more than one medication/or therapy. Slices in gray indicate no additional medication was taken

Sequences of treatment regimens in MDCD data are shown in the sunburst figure presented in Figs. 1. The figure emphasizes that although behavior therapy was the most common first-line intervention, all pharmacotherapies were very diverse with no one class of medication (or a single drug) that dominated. The first line of treatment is represented with the first ring; behavioral therapy (brown color) accounted for more than half of the ring. Moving from the inner ring to outer rings, we can see that 29.1% individuals received only behavioral therapies for the 3 years of follow up. Second line treatment are dispersed and included multiple regimens of single and combination therapies. The most common treatment change for those initiating behavioral therapies was a switch to centrally acting sympathomimetics or to imidazoline receptor agonists or to a combination therapy that adds these medications to behavioral therapy. For those who initiated treatment on a pharmacotherapy, the most common change was to add behavioral therapy. In Optum, the same trends were observed.

## Discussion

This study provides real world evidence to improve the understanding of the current landscape of both treatment and comorbidities of patients with ASD. We characterized patients’ comorbidity burden, and the treatment pathways based on more than 35 thousand ASD patients using observational data from routine clinical practice. Overall patients with ASD were found to have a high level of comorbidity burden with significant cooccurrence of ADHD and anxiety, especially among adolescents. Of the total cohort of patients with ASD in both data sources, around 87% received an intervention; with more than 70% receiving a behavior therapy and around 60% receiving at least one of the pharmacotherapies included in the study. Our data indicate that combination therapy and treatment changes are common among patients with ASD.

Much of the prior research examining treatment patterns used cross sectional data and focused on either behavioral and group interventions or a few classes of selected medications. This study provides a more comprehensive picture of treatments received and treatment changes over the short and long term.

Behavioral interventions are considered the current gold-standard treatment for behavioral symptoms associated with ASDs (Subramanyam et al., 2019). We found that at least 70% received such therapies at any time during the follow up period. We also found that approximately 50% of patients with ASD initiated treatment with behavioral therapy and up to 30% continued to only receive behavioral therapies; the rest moved on to either add or switch to pharmacotherapies. Recent studies have reported slightly inconsistent results on the utilization of behavioral therapy among patients with ASD; Zablotsky et al., (2015) reported that 19% of ASD children in their study were found to not receive any ASD related school or community services. Another cross-sectional study analyzed data from the 2016 National Survey of Children’s Health (NSCH) and found that 34.4% of patients with ASD were not receiving behavioral treatment (Xu et al., 2018). In contrast, Monz et al., (2019) reported that almost all children (96.0%) received behavioral therapy in a US‐wide sample of ASD children obtained from the Simons Foundation Powering Autism Research for Knowledge (SPARK) cohort. Such inconsistencies may be due to differences in the socioeconomic and demographic factors (including age) of the included population in each study. For example, insured children are more likely to receive behavioral treatment than those who are not (Xu et al., 2018). Additionally, our study may not have captured group level behavioral interventions mediated by schools or other community organizations.

There are limited pharmacotherapy treatment options to improve the symptoms associated with ASD (Subramanyam et al., 2019), with only two medications currently approved by the FDA (US Food & Drug Administration, 2021). Furthermore, neither of the approved medications is designed to address the core symptoms of social dysfunction and repetitive behaviors. However, consistent with our findings, studies have reported that, psychotropic medications are widely used by patients with ASD (Masi et al., 2017) to treat associated conditions such as ADHD, behavioral problems, anxiety, depression and seizures. Using data from a national sample of 2077 children diagnosed with ASD, Zablotsky et al., (2015)) reported that 52.7% of children used at least one medication. The patterns reported by Zablotsky et al. were similar to what we observed in the current study; with stimulants being the most common (32.6%), followed by anxiety and mood stabilizers (22.8%) and anti-depressants (17.9%). The same study reported that combination therapy was 30.3% in the study sample. While the use of these treatments (single and in combination) is prevalent, there is still minimal evidence to support the benefit of most these treatments in improving ASD core and associated symptoms.

Our data indicate that the majority of patients with ASD starting on any treatment make subsequent change(s). Moreover, among those whose treatment is changed, there is a decrease in behavioral interventions uptake. The frequent change of treatments may be explained by the complexity and the heterogeneity of the ASD prognosis and the need to try multiple approaches to address emerging core and associated ASD symptoms. Our data also indicate a high level of heterogeneity in treatments among individuals, even at initiation. Such heterogeneity may be explained by the genetic, environmental, cognitive, clinical (presence of comorbidities) and social heterogeneity of patients with ASD themselves (Masi et al., 2017). Finally, the lack of evidence to support the use of a particular medication or a class of medication (or a combination) to treat core symptoms and reliance on treating co-morbidities may be another factor contributing to the observed diversity in medication use both across and within individual patients.

We used two different national claims data bases in this study. The burden of ASD comorbidities, specifically ADHD and anxiety were higher in the MDCD data. This may be explained by differences in the underlying populations. MDCD typically represents lower socioeconomic status population with generally higher morbidities, while Optum represents a commercially (typically employed) insured population. However, overall, we observed consistent trends in treatments utilization across the two ASD cohorts.

There are several limitations of this research. First, patients with ASD were identified using diagnoses codes which are not a perfect tool. To mitigate for this drawback, we used a previously published algorithm for identifying ASD in claims data which achieved acceptable validity (Burke et al., 2014). Because the algorithm requires two outpatient diagnoses within a year, there is less of a chance of falsely classifying a patient as having ASD due to a rule-out or misdiagnosis as opposed to requiring only a single diagnosis. However, we do not capture patients who don’t fit this criterion. This is a trade-off we deliberately made to improve certainty that we only included true ASD patients. Second, we did not examine the average time patients were actively receiving each treatment, or how long patients may have been with no treatment between switching from one treatment to the next, as it was outside of the scope of this research. Third, there is no diagnosis associated with prescription claims, so the link between the medication received with a given co-morbidity/ associated symptoms (or an effort to treat core symptoms) cannot be established. To mitigate against attributing treatment to conditions not associated with ASD, we required treatment to occur at the time of or following the first diagnosis of ASD; however, this does not guarantee that treatments could not have been for other conditions where treatment was begun following a patient’s first ASD diagnosis. Additionally, the pharmacy claims are a record of medication dispensed to a patient which are not necessarily taken by the patient. Forth, our study required three-years of continuous observation following the index ASD diagnosis. This follow up requirement was chosen to capture sufficient follow up across the population to allow us to see multiple lines of therapy and various treatment changes. This approach likely excluded a portion of individuals with ASD. However, the alternate approach of allowing a shorter follow-up period would have failed to fully capture the natural history of treatment patterns over time. Lastly, the healthcare databases used do not capture the severity of the autism. Changes in autism severity could partially explained the great variety of treatments observed.

This study provides a detailed reflection of real-world comorbidity burden and behavior therapy services and pharmacotherapy practices for the treatment of ASD in the United States. Understanding the clinical complexity patients with ASD in the real world and how are they being managed is an important step to understanding unmet therapeutic needs and in improving care.

## Supplementary Information

Below is the link to the electronic supplementary material.

Supplementary file1 (docx 52 kb)
