# Supplementary material for: Autism: Comorbidities and Treatment Patterns in the Real World, a Retrospective Cohort Study Among Children, Adolescents and Adults Newly Diagnosed with Autism
Source: J Autism Dev Disord. 2021 Oct 8;52(10):4311–20. doi: 10.1007/s10803-021-05289-x (PMC9508210; doi:10.1007/s10803-021-05289-x)
Supplement: Supplementary file 1 — Supplementary file1 (docx 52 kb) [file 10803_2021_5289_MOESM1_ESM.docx]

**Supplemtary material for**

**Title:** **Autism: Comorbidities and treatment patterns in real world data**

Table 1. Behavioral and educational interventions procedure codes

| **Procedure** | **Code** | **Vocabulary** |
| --- | --- | --- |
| Therapeutic exercises to develop strength and endurance, range of motion and flexibility | 97110 | CPT4 |
| Therapeutic activities, direct (one-on-one) patient contact (use of dynamic activities to improve functional performance) | 97530 | CPT4 |
| Prolonged evaluation and management or psychotherapy service(s) | 99354, 99355 | CPT4 |
| Mental health service plan development by non-physician | H0032 | HCPCS |
| Mental health clubhouse services | H2030- H2030 | HCPCS |
| Psychologic AND/OR psychiatric procedure AND/OR service | 108310004* | SNOMED |
| Speech therapy | 5154007* | SNOMED |
| Language promotion therapy | 311602008* | SNOMED |
| Psychosocial procedure | 72353004* | SNOMED |
| Counseling | 409063005* | SNOMED |
| Art therapy | 6515300* | SNOMED |
| Depression management program | 401174001* | SNOMED |
| Nutrition therapy | 386373004* | SNOMED |
| Psychotherapy | 75516001* | SNOMED |
| Social prescribing for mental health | 515721000000104* | SNOMED |
| Occupational therapy | 84478008* | SNOMED |

Table 2. treatments considered in the treatment pathway analysis and it’s corresponding billing code

| **Category** | **Specific treatments considered in the treatment pathway analysis** |
| --- | --- |
| **Behavioral and educational** | All procedures listed in table 1 above grouped together. |
| **Brian stimulation approaches** | Transcranial Magnetic Stimulation (TMS) and transcranial Direct Current Stimulation grouped together-  CTP code : 0160T- 0161T, 90868, 90867,  SNOMED: 271406004* |
| **Medications** | Selective serotonin reuptake inhibitors |
|  | Serotonin–norepinephrine reuptake inhibitor |
|  | Tricyclic Antidepressant |
|  | Monoamine oxidase inhibitor |
|  | Other antidepressants |
|  | Antipsychotics- (excluding other Antipsychotics) |
|  | Anxiolytics |
|  | Anticonvulsant |
|  | Centrally acting sympathomimetics- (including Methylphenidate+ Amphetamine and others) |
|  | Hypnotics and sedatives |
|  | NMDA receptor antagonist (Memantine+Amantadine) |
|  | Acetylcysteine |
|  | Pentoxifylline |
|  | Riluzole |
|  | Celecoxib |
|  | Simvastatin |
|  | Baclofen |
|  | [Imidazoline receptor agonists](https://www.whocc.no/atc_ddd_index/?code=C02AC&showdescription=no) (Guanfacine and Clonidineand others) |
|  | other Antipsychotics (Risperidone + Aripiprazole) |
|  | Oxytocin |
